# On the substrate turnover rate of NBCe1 and AE1 SLC4 transporters: structure-function considerations

**DOI:** 10.3389/fphys.2024.1474628

**Published:** 2025-01-13

**Authors:** Alexander Pushkin, Liyo Kao, Hristina R. Zhekova, Rustam Azimov, Natalia Abuladze, Xuesi M. Shao, D. Peter Tieleman, Ira Kurtz

**Affiliations:** ^1^ Department of Medicine, David Geffen School of Medicine, University of California, Los Angeles, Los Angeles, CA, United States; ^2^ Centre for Molecular Simulation, Department of Biological Sciences, University of Calgary, Calgary, AB, Canada; ^3^ Department of Neurobiology, David Geffen School of Medicine, University of California, Los Angeles, Los Angeles, CA, United States; ^4^ Brain Research Institute, David Geffen School of Medicine, University of California, Los Angeles, Los Angeles, CA, United States

**Keywords:** ion turnover rate, bicarbonate, carbonate, transport, renal tubular acidosis, NBCe1-A, AE1

## Abstract

A transport protein’s turnover rate (TOR) is the maximum rate of substrate translocation under saturating conditions. This parameter represents the number of transporting events per transporter molecule (assuming a single transport site) per second (s). From this standpoint, a transporter’s TOR is similar to an enzyme’s catalytic constant. Knowledge of a transporter’s TOR allows comparison of the transport capacity of various transporters at the molecular level as well as the total transport per cell and whole organ levels. Despite this, there is currently a very limited number of transporters, for which TOR has been determined experimentally. In the SLC4 transporter family of CO_3_^2−^/HCO_3_^−^ transporters, erythrocyte AE1 (eAE1; SLC4A1) is the only member, for which TOR has been determined (∼50,000 s^−1^). Whether other SLC4 family members have similar TOR values is currently unknown. Here we report TOR measurements of the electrogenic Na^+^-CO_3_^2−^ cotransporter NBCe1-A (SLC4A4) and the kidney specific AE1 splice variant, kAE1, that play important roles in renal bicarbonate absorption and are mutated in proximal and distal renal tubular acidosis respectively. We have also remeasured the eAE1 TOR value for comparison. NBCe1-A had a TOR value of ∼30,400 s^−1^ whereas kAE1 and eAE1 had significantly higher values (62,000 s^−1^ and 60,500 s^−1^ respectively). We modeled the inward-facing (IF) conformation of NBCe1-A to determine conformational changes during its transport cycle. Comparison of this IF model with our previously determined cryoelectron microscopy (cryoEM) outward-facing (OF) conformation structure, demonstrates that NBCe1-A has an elevator-type transport mechanism with a small vertical ∼5 Å shift of the ion coordination site as we have previously shown for AE1. We speculate that this very small vertical movement plays an important role in contributing to the very high TOR numbers of SLC4 transporters.

## 1 Introduction

Evaluation of the functional roles that transporters play in various organs, tissues and single cells often requires knowledge of their maximal transport rates. The transporter turnover rate (TOR) representing the maximum rate of substrate translocation supported by a single transporter molecule under substrate saturating conditions (Stein, 1968; Brahm, 1977; Karlish and Stein, 1982; Stein, 1986; Beckstein and Naughton, 2022; Wright and Secomb, 2022; Zhang and Wright, 2022), has significant advantages. Specifically, TOR is equal to the number of substrate transport events mediated by a single transporter site per unit of time. For homo-oligomers with individual functional monomeric subunits, the TOR number refers to the function of each monomer. From this standpoint, a transporter’s TOR is similar to an enzyme’s k_cat_ catalytic constant (Beckstein and Naughton, 2022). Knowledge of transporter’s TOR value allows comparison of the transport capacity of various transporters at the molecular level as well as the calculation of the total rate transport per cell. Despite these advantages, there is currently a very limited number of membrane transporter proteins for which TOR values have been determined (Brahm, 1977; Dixon et al., 1987; Taglicht et al., 1991; Gu et al., 1994; Siczkowski and Ng, 1995; Pos and Dimroth, 1996; Smith et al., 2004; Cha et al., 2009; Gonzales et al., 2007; Maryon et al., 2013; Wöhlert et al., 2015; Severance et al., 2017; Wang et al., 2022; Zhang and Wright, 2022).

Of the minority of transporters (uniporters, symporters and exchangers) whose TOR numbers have been measured, there is a wide range from ∼0.02 s^−1^ to a maximum of ∼50,000 s^−1^ ((Brahm, 1977; Dixon et al., 1987; Taglicht et al., 1991; Gu et al., 1994; Siczkowski and Ng, 1995; Pos and Dimroth, 1996; Smith et al., 2004; Cha et al., 2009; Gonzales et al., 2007; Maryon et al., 2013; Wöhlert et al., 2015; Severance et al., 2017; Wang et al., 2022; Zhang and Wright, 2022). Among these transporters, eAE1 has the highest known value of ∼50,000 s^−1^ (Brahm, 1977) that was proposed to be associated with its key role in mediating erythrocyte CO_2_ transport from tissues to the lungs (Reithmeier et al., 2016). eAE1 belongs to the SLC4 CO_3_^2−^/HCO_3_^−^ transporter family of 10 membrane proteins and is the only member for which TOR has been determined (Brahm, 1977). The majority of members of this family mediate the transport of CO_3_^2−^ or HCO_3_^−^ in a Na^+^ and/or Cl^−^–dependent/independent manner and are expressed in multiple tissues where they play an important role in both intracellular/extracellular pH maintenance and ion homeostasis (Parker and Boron, 2013; Kurtz, 2014; Reithmeier et al., 2016; Kurtz and Schwartz, 2023; Lee et al., 2023). SLC4 family members have a high amino acid sequence homology despite their differing transport properties and modes (symporter versus exchanger): 1) Na^+^-independent electroneutral Cl^−^/HCO_3_^−^ exchange (AE1, AE2, AE3), 2) electroneutral Na^+^–HCO_3_^−^ cotransport (NBCn1 and NBCn2), 3) electrogenic Na^+^–CO_3_^2−^ cotransport (NBCe1 and NBCe2), and 4) electroneutral Na^+^−CO_3_^2^^−^/Cl^−^ exchange (NDCBE) and (K^+^/Na^+^)–CO_3_^2−^/Cl^−^ exchange (AE4). SLC4A11, which has significantly less homology with other members, transports H^+^/OH^−^ (Parker and Boron, 2013; Zhang et al., 2015; Kao et al., 2020).

All structurally characterized SLC4 transporters are homodimers with each monomer consisting of a gate (scaffold) and core (transport) domain (Arakawa et al., 2015; Huynh et al., 2018; Wang et al., 2021; Zhekova et al., 2022; Zhang et al., 2023; Lu et al., 2023). The gate domain plays a key role in their dimerization and do not move during the transport cycle, whereas the core domain possesses the substrate coordination site, which shifts vertically together with the bound substrates during the transport cycle (Zhekova et al., 2022; Lu et al., 2023; Zhang et al., 2023). Due to this vertical shift of the substrate coordination site, the SLC4 transporters belong to the so-called elevator-type transporters (Garaeva and Slotboom, 2020).

In the kidney, proximal tubule NBCe1-A which is now thought to transport Na^+^ coupled to CO_3_^2−^ rather than HCO_3_^−^ (Huynh et al., 2018; Kurtz and Schwartz, 2023; Lee et al., 2023) and kAE1, the collecting duct AE1 Cl^−^/HCO_3_^−^ exchanger isoform (Kollerton-Jons et al., 1993) play key roles in renal bicarbonate absorption. Mutations in these proteins cause proximal and distal renal tubular acidosis, respectively (Kurtz, 2018; Wagner et al., 2023). Given the aforementioned considerations and recently demonstrated identity of the structural fold of several SLC4 transporters (Arakawa et al., 2015; Huynh et al., 2018; Wang et al., 2021; Zhekova et al., 2021; Zhekova et al., 2022; Wang et al., 2022; Zhang et al., 2023; Lu et al., 2023; Jian et al., 2024), we sought to determine whether these renal disease-causing family members have a similarly high TOR analogous to eAE1 or whether the high TOR value of eAE1 is unique among all known membrane transport proteins. NBCe1-A, kAE1 and eAE1 transport was measured using intracellular pH (pH_i_) determined base flux. NBCe1-A transport was also measured using whole cell patch clamp assays. For each transporter, a V5 epitope was inserted into an exposed extracellular loop so that the expression of the number of transporters could be accurately quantitated using V5 epitope protein standards.

## 2 Materials and methods

### 2.1 NBCe1-A Na^+^-driven CO_3_^2−^ flux

HEK293 cells were grown on glass polyethyleneimine (PEI) coated 25 mm coverslips and transiently transfected with human wt-NBCe-1A containing a V5 tag inserted into extracellular loop 3 (EL3; 606–LPTMGKPIPNPLLGLDSTSSTD–627) or an empty pcDNA3.1(+) expression vector (mock transfected) using Lipofectamine 2000 according to the manufacturer’s protocol. The background mock transfected flux was subtracted from the data obtained in NBCe1-A transfected cells to determine the NBCe1-A specific flux. Table 1 depicts the solutions used in these experiments (chemicals were from Sigma-Aldrich or Fisher Scientific). 24 h following transfection, the coverslips with the transfected cell monolayers were placed into a custom flow-through chamber on the stage of a modified custom-built dual excitation microscope-fluorometer (Kurtz, 1987). In brief, the device currently consists of a modified Zeiss ICM microscope (Carl Zeiss, Inc.) that enables the monitoring of the fluorescence of an intracellular dye excited simultaneously by 2 different wavelengths. In the case of an excitation ratiometric dye such as BCECF, measuring individual wavelengths prevents dye loss or bleaching from affecting the excitation ratio. FLED light sources (Sutter Instruments), or 75-W xenon arc lamps are used as excitation sources with the appropriate bandpass filters. For the current study, BCECF loaded cells were excited simultaneously at 440-nm (pH independent wavelength) and 500-nm (pH dependent wavelength). The two excitation light sources were attenuated with neutral density filters and modulated at different frequencies using five-slot choppers (Stanford Research Systems) coupled to the output of each light source. The excitation beams initially at right angles to each other were combined into a single path with a dichroic mirror (transmits 500-nm wavelength and reflects 440-nm wavelength) that interfaces with the microscope. Both modulated excitation wavelengths were then reflected to the cell monolayer and the emitted 530-nm fluorescence BCECF emission was detected in an epifluorescence configuration by a photomultiplier tube (Thorn EMI) driven by a HV series 477 power supply (Brandenburg). The emitted 530-nm signal (which is also modulated at the same two frequencies as the excitation sources) is resolved into its 440- and 500-nm components using two lock-in amplifiers (Model SR510; Stanford Research Systems). The two fluorescence intensities were digitized using a custom designed LabMaster data acquisition system (Scientific Solutions) and the 500/440 nm excitation ratio as a function of time was displayed in real time during each experiment.

**TABLE 1:** Intracellular pH measurement solutions.

	1	2	3	4
NaCl			115	
TMACl	140	115		
TMAGluconate				115
CaCl_2_	1	1	1	
MgCl_2_	1	1	1	
CaGluconate				7
MgGluconate				2
K_2_HPO_4_	2.5	2.5	2.5	2.5
Dextrose	5	5	5	5
HEPES	5			
NaHCO_3_			24	
TMAHCO_3_		24		24
pH	7.4	7.4	7.4	7.4

All values are in (mM); all solutions contained 30 μM EIPA, and bicarbonate-containing solutions were bubbled with 5% CO_2_.

The cells were loaded with the fluorescent pH_i_ probe BCECF (esterified BCECF-AM; Life Technologies) in a Na^+^-free, Cl^−^-containing, HCO_3_^−^-free solution (solution 1) for ∼25 min at room temperature. Following BCECF loading, the fluorescence data (500 nm/440 nm excitation ratio; emission 530 nm) from ∼200 cells (20X objective; single field of view) was acquired every 0.5 s at 37°C and the data was calibrated (excitation ratio converted to pH) at the end of each experiment with valinomycin (Sigma-Aldrich) and nigericin (Sigma-Aldrich). Following dye loading and initially bathing the cells in solution 1, the cells were next switched to a Na^+^-free, Cl^−^-containing, HCO_3_^−^-buffered solution (solution 2). After a steady state, NBCe1-A mediated Na^+^-driven CO_3_^2^‾ flux was initiated by exposing the cells to a Na^+^-, Cl^−^-, and HCO_3_^−^-containing solution (solution 3). The initial rate of change of [H^+^_in_] (d[H^+^_in_]·dt^−1^) was measured in the first 10–15 s after exposing the cells to solution 3. CO_3_^2−^ flux was calculated by multiplying d[H^+^_in_]·dt^−1^ by the total cell buffer capacity (intrinsic buffer capacity βi plus the bicarbonate buffer capacity (βHCO_3_)). The experimental results for NBCe1-A were obtained from 12 coverslips (3 cell batches).

### 2.2 kAE1 and eAE1 Cl^−^-driven HCO_3_^−^ flux

The cells were transfected with human wt-kAE1 containing a V5 tag inserted into extracellular loop 3 (EL3; 490–YNVLGKPIPNPLLGLDSTMVPK–511), human wt-eAE1 containing a V5 tag inserted into extracellular loop 3 (EL3; 555–YNVLGKPIPNPLLGLDSTMVPK–576) or an empty pcDNA3.1(+) expression vector (mock transfected). The mock transfected flux was subtracted from the data obtained in AE1 transfected cells to determine the AE1 specific flux. Cl^−^-driven HCO_3_^−^ flux was measured 24 h following transfection. The cells were loaded with BCECF as described above and initially bathed in solution 1. After a steady state the cells were bathed in a Na^+^-free, Cl^−^-containing, HCO_3_^−^-buffered solution (solution 2; Table 1). After a steady state, kAE1 and eAE1 mediated Cl^−^-driven HCO_3_^−^ flux was induced by switching to a Na^+^-free, Cl^−^-free, HCO_3_^−^-buffered solution (solution 4; Table 1). The Cl^−^-driven HCO_3_^−^ flux was measured in the first 10–15 s after bathing the cells in solution 4 and calculated as (βi + βHCO_3_)·d[H^+^_in_]·dt^−1^. The experimental results for kAE1 were obtained from 14 coverslips (4 cell batches); and for eAE1 from 12 coverslips (4 cell batches).

### 2.3 Plasma membrane protein labeling of V5-tagged NBCe1-A, kAE1 and eAE1

24 h following transfection, plasma membrane proteins were biotinylated to detect the V5-tagged transporters according to the following protocol. The cells were grown on 6-well plates and the cells from one well (3.54 ± 0.16 × 10^6^ cells per well; n = 24 wells; 6 cell batches) were washed with PBS (pH 8.0) and resuspended in PBS (pH 8.0) with 1.1 mM sulfo-NHS-SS-biotin (Thermo Fisher Scientific) for 30 min on a rotator. The reaction was stopped with 50 mM Tris (pH 8.0) containing 140 mM NaCl. The cells were then washed with PBS (pH 8.0) and lysed in 500 μL on ice in 10 mM Tris-HCl (pH 7.5), 150 mM NaCl, 5 mM EDTA (Sigma-Aldrich), 0.5% sodium deoxycholate (Thermo Fisher Scientific), 1% (vol/vol) Igepal (Sigma-Aldrich), and protease inhibitor cocktail (Roche Life Sciences). Following centrifugation at 16,000 *g* for 10 min at 4°C, the 500 μL supernatant was collected and incubated with 30 μL streptavidin-agarose resin (Thermo Fisher Scientific) for 4 h on a rotator at 4°C. After brief centrifugation to pellet the resin and washing with the lysis buffer, bound proteins were eluted for 5 min at 60°C with 2 × SDS buffer and 2% β-mercaptoethanol (EMD Millipore).

### 2.4 Quantitation of plasma membrane transporter number

A V5 peptide standard curve was generated using a Multiple tags (GTX130343-pro, GeneTex) peptide that contained several tags including the V5 tag (Zhang and Wright, 2022). Various amounts of V5 protein from 0.0625 ng (1.36 fmol) to 1 ng (21.7 fmol) were run on the same 4-15% polyacrylamide gel with biotinylated V5-tagged NBCe1-A, kAE1 and eAE1. The samples were then transferred to polyvinylidene difluoride (PVDF) membranes (GE Healthcare) and probed with a V5 monoclonal antibody (R960-25, Thermo Fisher Scientific; dilution 1:10,000) in TBSTM buffer: 20 mM Tris-HCl (pH 7.5) containing 137 mM NaCl, 0.1% (vol/vol) Tween 20% and 5% nonfat milk (w/vol) at room temperature for 1 h. The blots were then washed with TBST and labeled with Peroxidase AffiniPure Donkey Anti-Mouse IgG (H + L) secondary antibody (Jackson ImmunoResearch; 1:10,000 dilution) in TBSTM at room temperature for 1 h. Following washing with TBST, ECL Western blotting Detection Reagent (GE Healthcare) was used for detection. Using this approach, the complete set of V5 standards and plasma membrane transporter protein could be detected on the same x-ray film with the same exposure at the same time. Previous studies have shown that NBCe1-A and AE1 monomers function independently (Macara and Cantley, 1981a; Macara and Cantley, 1981b; Kao et al., 2008). To obtain the total number of transporting monomers, the intensity of both monomeric and dimeric transporter bands was measured, combined and compared to the intensity of the V5 standard curve bands using the histogram function in Adobe Photoshop.

### 2.5 Measurement of cell number and volume

Cell number and cell diameter were measured immediately following cell re-suspension using an automated cell counter (Cellometer Auto T4; Nexcelom Bioscience). Assuming the cells were approximately spherical when re-suspended, cell volume could be calculated where volume = 4/3πr^3^.

### 2.6 Correction for transporter expression efficiency

Since protein expression following transient transfection protocols is not 100% efficient, to correct for this effect, 24 h post transfection of V5-tagged NBCe1-A, kAE1 or eAE1, the percentage of cells expressing the plasma membrane tagged transporters was assessed fluorescently by immunocytochemistry using the V5-specific monoclonal primary antibody (PBS; 1:100 dilution; 30 min at room temperature) followed by labeling with Cy3 secondary antibody (PBS; 1:500; 30 min at room temperature; Jackson ImmunoResearch Laboratories Inc.) Cell nuclei were identified by treating the cells with methanol and then labeling with Hoechst dye for 10 min (PBS; 1:500; Sigma Aldrich). A SPOT RT sCMOS camera (Spotimaging) coupled to a Nikon Microphot-FXA epifluorescence microscope (20x objective) was used to capture transmission and fluorescence images of the cells transfected with either V5-tagged NBCe1-A, kAE1 or eAE1. The digitized images were displayed in Adobe Photoshop. Approximately 500 cells were analyzed per field (5 fields per coverslip) and the percentage of the total cells expressing the plasma membrane V5-tagged transporters was determined using in at least 3 separate transfection experiments for each construct. The expression efficiency determined for the NBCe1-A, kAE1 and eAE1 constructs assessed randomly throughout the study (43.7 ± 0.18 percent; n = 9) was consistent with the data obtained separately from cells that were transfected at the same time the functional measurements were obtained (46.6 ± 0.98 percent; n = 11).

### 2.7 TOR calculation

Given that in the pH_i_ base transport experiments, the transporter flux is measured in units of mM·s^-1^, following mock-transfected cell flux subtraction, the transporter specific flux was converted to mol·s^-1^·cell^-1^ by multiplying by the measured cell volume per cell. In the immunoblot experiments, after determining the total number of moles of plasma membrane transporter protein monomers, the results were converted to units of mol·cell^−1^ by dividing by the number of cells from which the transporter biotinylated protein samples loaded onto the gels were derived. The transporter flux terms and the calculation of the transporter monomer number per cell were also corrected for transporter protein expression efficiency prior to calculation of the final TOR values. TOR (s^−1^) was then calculated for each transporter by dividing the transporter flux (mol·s^−1^·cell^−1^) by the plasma membrane transporter monomer number (mol·cell^−1^).

### 2.8 NBCe1-A transport current and TOR-dependence on membrane voltage

Whole cell patch clamp measurements of the NBCe1-A transport currents were performed as previously described (Zhu et al., 2013; Shao et al., 2014; Huynh et al., 2018) with the following modifications. To identify cells expressing V5-tagged NBCe1-A, 24 h after transfection the cells were re-seeded onto cell culture dishes (Delta T TPG Non-Heated Culture Dishes; Bioptechs Inc.). After 1 h, the cells were labeled with the V5 monoclonal antibody (1:100 dilution) for 30 min followed by the Cy3 secondary antibody (1:500; 30 min). The cell culture dish was placed into a chamber on the stage of a fluorescent microscope (Axioskop 2 FS plus; Carl Zeiss) to image the cells under transmitted light and identify fluorescently labeled cells expressing NBCe1-A that could be patched. The cells were continuously perfused at a flow rate of ∼2 mL·min^−1^ (room temperature). Cells expressing NBCe1-A were then chosen for electrophysiological measurements using a MultiClamp 700B patch amplifier (Molecular Devices) and borosilicate glass patch pipettes with a tip diameter of 1–1.5 μm (tip resistance 4–6.5 MΩ). To ensure stable electrode potentials during recordings, a microagar salt bridge containing 2 M KCl in the patch pipet was utilized (Shao and Feldman, 2007). The signal was low-pass filtered at 400 Hz and sampled at 2 kHz using the pClamp software (Clampex 10, Molecular Devices). Whole cell capacitance and series resistance were determined using the auto-whole cell capacitance and series resistance compensation. The series resistance was typically compensated 80% (correction and prediction). In the first group of experiments, NBCe1-A mediated whole cell currents were measured using solutions that approximated the intracellular-extracellular Na^+^ and CO_3_^2-^ concentration gradients present during the NBCe1-A mediated pH_i_ base transport measurements. The NBCe1-A transport currents and TOR values were also determined at various membrane voltages. The solutions used in these experiments are shown in Table 2 (chemicals were from Sigma-Aldrich). The cells were patched with a Na^+^-free, Cl^−^-, and HCO_3_^−^-containing, pH 6.90 solution (solution 1). The cells were bathed in a Na^+^-free, Cl^−^-, and HCO_3_^−^-containing, pH 7.4 solution (solution 2). After a steady state, the bath solution was then changed to a Na^+^-, Cl^−^-, and HCO_3_^−^-containing, pH 7.4 solution (solution 3) in the absence or presence (solution 4) of the NBCe1-A inhibitor tenidap (0.2 mM; Santa Cruz Biotechnology) (Kao et al., 2008) in a paired fashion. Steady-state currents were measured with a holding potential of −55 mV and a series of 400-ms voltage pulses from −100 to +40 mV. To determine the TOR values, the tenidap-inhibitable transport currents in units of pA·cell^−1^ were converted into units of mol·s^−1^·cell^−1^ using Faraday’s constant (96,485 C/mol) where flux in mol·s^−1^·cell^−1^ = current in pA·cell^−1^ × 10^−12^/96,485 (Fuster et al., 2008). These values were then divided by the cell plasma membrane transporter monomer number (mol·cell^−1^). The experimental results were obtained from 9 cell culture dishes (3 cell batches).

**TABLE 2:** Patch clamp solutions.

	1	2	3	4	5	6	7
NaCl			115	115			
CsCl		10	10	10		9	9
TMACl		120	5	5			
TEACl	10				10		
CsMeSO_3_	40						
NaGluconate						95	95
NaOH					95		
CsOH	43.5	5	5	5	10		
CaCl_2_	1.0	1.5	1.5	1.5	1.0	1.5	1.5
HEPES	100	10	10	10	10	10	10
Choline-HCO_3_	7.67	24					
NaHCO_3_			24	24	24	24	24
EGTA	10				10		
Mannitol						50	50
Tenidap				0.2			0.2
Gluconic Acid					70		
TMAOH						5	5
pH	6.9	7.4	7.4	7.4	7.4	7.4	7.4

All values are in (mM).

Pipet solutions: 1 and 5.

Bath solutions: 2,3,4,6 and 7.

To determine the dependence of the TOR values on the membrane voltage *per se* additional studies were done in the absence of Na^+^ and CO_3_^2−^ pipet to bath concentration gradients. The Na^+^ concentration in both the pipet (solution 5) and bath (solution 6) was 119 mM with an estimated CO_3_^2−^ concentration of 36.7 μM. Currents were measured in a paired fashion in the absence or presence of the NBCe1-A inhibitor tenidap (0.2 mM, solution 7). To minimize the NBCe1-A transport mediated changes in the patch pipet solution due to ion flux driven by multiple changes in the membrane voltage per cell, the number of voltage steps was decreased. Accordingly, steady-state currents were measured during whole cell patch clamp using a holding potential of 0 mV and 400-ms voltage pulses and using separate cells (one cell/dish) for one of the following voltage steps: −40 to 0 to +40 mV (n = 9 from 3 cell batches); −30 to 0 to +30 mV (n = 6 from 3 cell batches); −20 to 0 to +20 mV (n = 7 from 3 cell batches); −10 to 0 to +10 mV (n = 12 from 4 cell batches). The tenidap-inhibitable transport currents were converted into units of mol·s^−1^·cell^−1^ as described in section 2.7 above, and the values were then divided by the cell plasma membrane transporter monomer number (mol·cell^−1^) to determine the respective TOR values.

### 2.9 Modeling of the NBCe1-A IF state

The IF model of NBCe1-A was generated with the SwissModel server (Waterhouse et al., 2018) using the recently published AE2 IF conformation structure (PDB code 8GV9) (Zhang et al., 2023) as a template. To assess the OF to IF conformational changes, the OF and IF NBCe1-A structural models were aligned with respect to their rigid gate domains whose geometry does not undergo big changes during the transport process (Zhekova et al., 2022). The shift of the ion coordination site was calculated by comparison of the OF and IF position of the center-of-mass (COM_S1_) calculated from the backbone atoms of a set of residues (S483-P487, D754, T758, and V798-V802) that have been previously identified as part of ion coordination site (site S1) in OF NBCe1 (Zhekova et al., 2021).

### 2.10 Statistics

One-way ANOVA followed by Tukey-Kramer HSD was used to compare multiple study group means. Study group data is shown as mean ± SEM and considered significantly different when *p* < 0.05.

## 3 Results

### 3.1 Base transport functional assays

**FIGURE 1:**
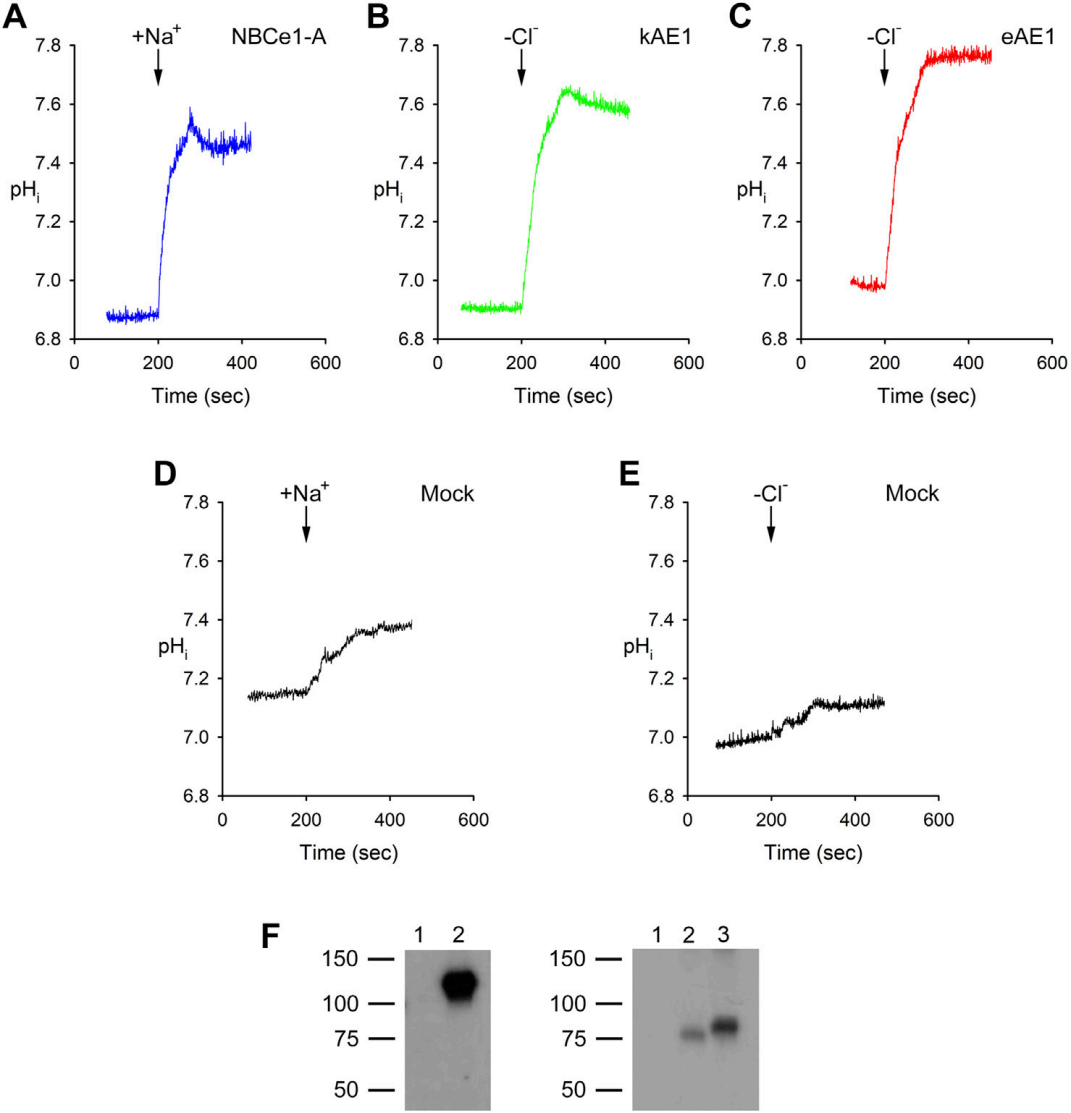
Typical pH_i_ traces in experiments assaying **(A)** NBCe1-A, **(B)** kAE1 and **(C)** eAE1 mediated transport. **(D, E)** Mock transfected. **(A, D)** After being bathed in a Na^+^-free, Cl^−^-containing, HCO_3_^−^-buffered solution (solution 2, Table 1), the cells were exposed (arrow) to a Na^+^-, Cl^−^-, and HCO_3_^−^-containing solution (solution 3, Table 1). The NBCe1-A mediated transport flux was calculated as detailed in Methods and materials, section 2.1. **(B, C, E)** The cells were initially bathed in a Na^+^-free, Cl^−^-containing, HCO_3_^−^-buffered solution (solution 2, Table 1) and then switched (arrow) to a Na^+^-free, Cl^−^-free, HCO_3_^−^-containing solution (solution 4; Table 1). kAE1 and eAE1 mediated transport flux was calculated as detailed in Methods and materials, section 2.2. **(F)** Experiments showing lack of expression of NBCe1-A, kAE1 and eAE1 in untransfected HEK293 cells used in the study. Left: Immunoblot of cell lysates: Lane 1, mock transfected. Lane 2, NBCe1-A transfected. Primary antibody: SC-162214 (Santa Cruz Biotechnology; 1:10,000 dilution); secondary antibody: Peroxidase AffiniPure Donkey Anti-goat IgG (H + L) secondary antibody (Jackson ImmunoResearch; 1:10,000 dilution). Right: Immunoblot of cell lysates: Lane 1, mock transfected. Lane 2, kAE1 transfected. Lane 3, eAE1 transfected. Primary antibody: PA5-141065 (Thermofisher Scientific; 1:10,000 dilution); secondary antibody: Peroxidase AffiniPure Mouse Anti-rabbit IgG (H + L) secondary antibody (Jackson ImmunoResearch; 1:10,000 dilution).

Typical representative pH_i_ base transport experiments for NBCe1-A, kAE1 and eAE1 are shown in Figure 1. In NBCe1-A transfected cells, a typical response to the addition of Na^+^ in the presence of bicarbonate is shown. Upon the addition of Na^+^, CO_3_^2−^ is driven into the cells resulting in an increase in pH_i_. For kAE1 and eAE1, the removal of external Cl^−^ in bicarbonate media drives HCO_3_^−^ into the cells causing an increase in pH_i_ as shown. Table 3 summarizes the NBCe1-A mediated cell CO_3_^2−^ flux and respective kAE1 and eAE1 mediated cell HCO_3_^−^ flux values.

**TABLE 3:** TOR value determination of NBCe1-A, kAE1 and eAE1.

NBCe1-A		
Flux (mol·s^−1^·cell^−1^)	PM monomers (mol·cell^−1^)	TOR (s^−1^)
2.40 ± 0.23 × 10−15	7.89 ± 0.90 × 10^−20^	30,422 ± 1,968
n = 12	n = 8	n = 12
kAE1		
Flux (mol·s^−1^·cell^−1^)	PM monomers (mol·cell^−1^)	TOR (s^−1^)
3.74 ± 0.40 × 10^−15^	6.00 ± 0.65 × 10^−20^	62,241 ± 4,692
n = 14	n = 8	n = 14
eAE1		
Flux (mol·s^−1^·cell^−1^)	PM monomers (mol·cell^−1^)	TOR (s^−1^)
3.75 ± 0.41 × 10^−15^	6.20 ± 0.53 × 10^−20^	60,491 ± 4,888
n = 12	n = 8	n = 12

The mean cell volume (L) in these experiments was 1.99 ± 0.14 × 10^−12^ (n = 35).

The flux values depicted are background (mock transfected) subtracted. For the NBCe1-A experiments, the background flux was 2.10 ± 0.06 × 10^−16^ (mol·s^−1^·cell^−1^) (n = 11, 3 cell batches). In the AE1 experiments the background flux was 2.91 ± 0.23 × 10^−16^ (mol·s^−1^·cell^−1^) (n = 7, 3 cell batches).

TOR, value comparisons: NBCe1-A vs. kAE1, *p* < 0.0001; NBCe1-A vs. eAE1, *p* < 0.0001; kAE1 vs. eAE1, p = NS.

### 3.2 Immunoblot analysis of plasma membrane monomer number

**FIGURE 2:**
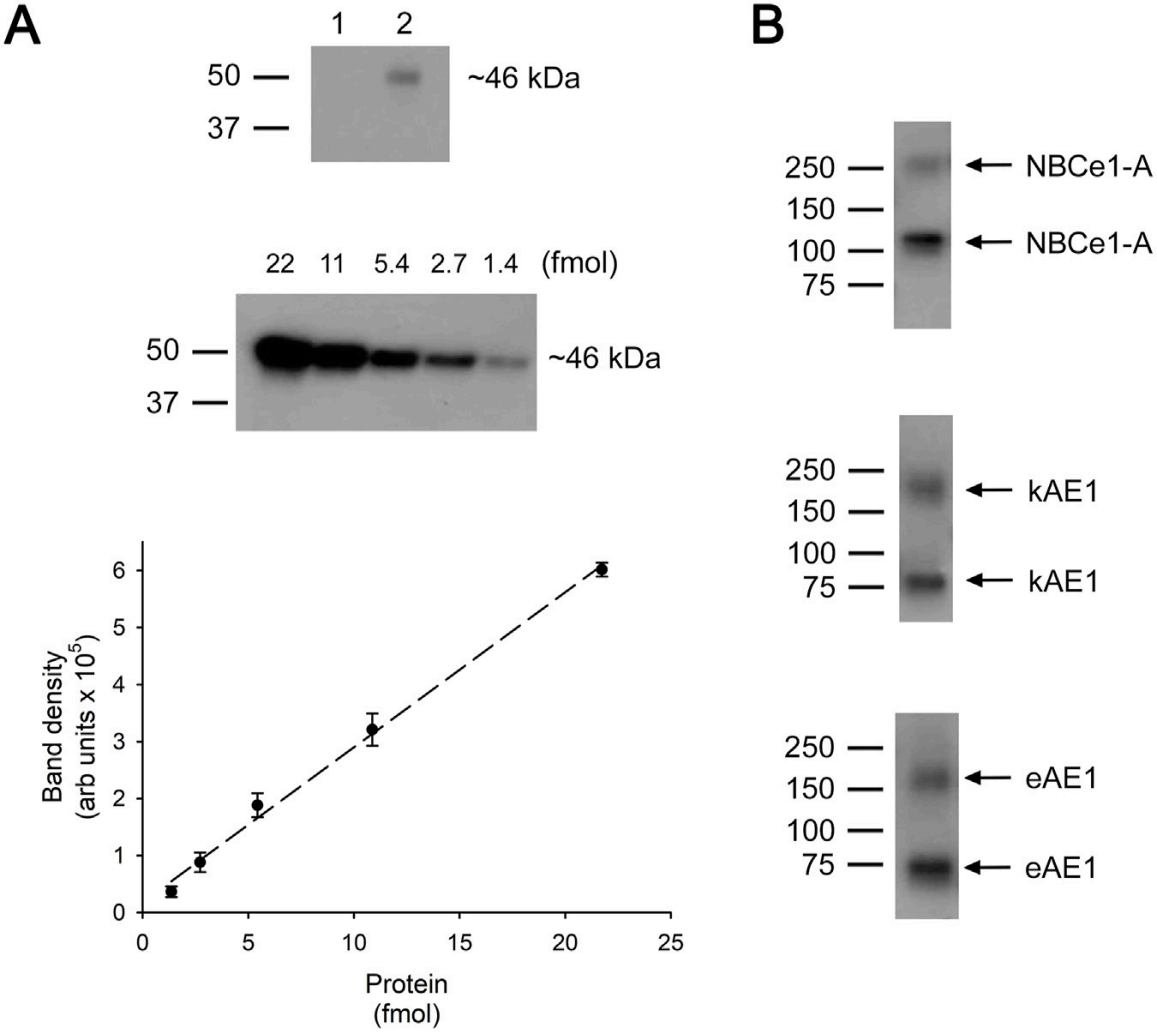
Quantitation of plasma membrane monomer number. **(A)** Top: Immunoblot: Lane 1, mock transfected cell lysate. Lane 2, V5 tag peptide standard (1.4 fmol). **(A)** Middle: Immunoblot using various amounts of V5 tag peptide standard. **(A)** Bottom: V5 tag peptide standard calibration curve. The data represents the mean ± SEM data of 8 separate experiments. Dotted line represents a linear fit to the data. **(B)** Immunoblots of biotinylated V5-tagged NBCe1-A, kAE1 or eAE1 loaded onto the same 4%–15% polyacrylamide gels as the V5 peptide standards. The upper band on each immunoblot represents dimers and the lower band monomers. To determine the total number of transporting monomers, the intensity of both monomeric and dimeric bands were measured and combined.

Figure 2 shows a representative immunoblot analysis of the V5 peptide standard curve and biotinylated V5-tagged NBCe1-A, kAE1 and eAE1. The mean cell plasma membrane (PM) monomer number for each transporter is summarized in Table 3.

### 3.3 NBCe1-A, kAE1 and eAE1 TOR values

Using the pH_i_ base transport data and cell membrane monomer values, the TOR number for each transporter was calculated and summarized in Table 3. The results show that all three transporters have exceedingly high TOR values in comparison to other known transporters. Moreover, both kAE1 and eAE1 mediate anion exchange at a TOR value that exceeds NBCe1-A symport by ∼2-fold.

### 3.4 NBCe1-A transport currents and TOR values

**FIGURE 3:**
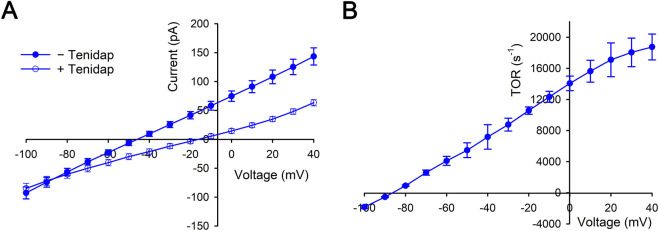
Whole cell patch clamp measurements of NBCe1-A transport currents in the presence of a fixed Na^+^, CO_3_^2−^ (estimated using the Henderson Hasselbalch equation) and pH gradient (intracellular Na^+^: 0 mM, CO_3_^2−^: 3.67 μM, pH 6.90; extracellular Na^+^: 139 mM, CO_3_^2−^: 36.7 μM, pH 7.40). In these experiments the pipet Na^+^ and CO_3_^2−^ concentrations (0 mM and 3.67 μM) approximated the intracellular Na^+^ and CO_3_^2−^ concentrations in the pH_i_ base transport studies (Figure 1) immediately prior to bathing the cells in a Na^+^-containing pH 7.4 solution. The intracellular (pH 6.9) and extracellular (pH 7.4) CO_3_^2−^ concentrations were estimated using the Henderson-Hasselbalch (H−H) equation. The data was derived from n = 9 separate whole cell patch clamp experiments. **(A)** Mean currents in the absence and presence of the NBCe1 inhibitor tenidap (paired fashion). **(B)** The tenidap-inhibitable currents were converted to flux (mol·s^−1^·cell^−1^) and using the cell membrane monomer values the TOR numbers were determined at each membrane voltage.

NBCe1-A mediates electrogenic flux unlike electroneutral kAE1 and eAE1 that is therefore dependent on both the transported ion gradients (Na^+^ and CO_3_^2−^) across the transporter and the membrane voltage. Using extracellular-intracellular Na^+^ and CO_3_^2−^ ion gradients that approximated the values during the pH_i_ base transport functional assays (Figure 1), the NBCe1-A TOR values were reassessed using an electrophysiologic approach (measuring the NBCe1-A transport current) while in addition determining the membrane voltage dependence (that could not be technically done in the pH_i_ base transport studies). NBCe1-A-mediated currents and TOR values at a fixed Na^+^, CO_3_^2−^ (estimated using the Henderson Hasselbalch equation) and pH gradient (intracellular Na^+^: 0 mM, CO_3_^2−^: 3.67 μM, pH 6.90; extracellular Na^+^: 139 mM, CO_3_^2−^: 36.7 μM, pH 7.40) are shown in Figure 3. The data in Figure 3A shows the mean currents in the absence and presence of the NBCe1 inhibitor tenidap. The tenidap-inhibitable currents at various membrane voltages were converted to flux (mol·s^−1^·cell^−1^; see Methods and materials 2.8) and using the cell membrane monomer values, the TOR numbers were calculated as depicted in Figure 3B.

**FIGURE 4:**
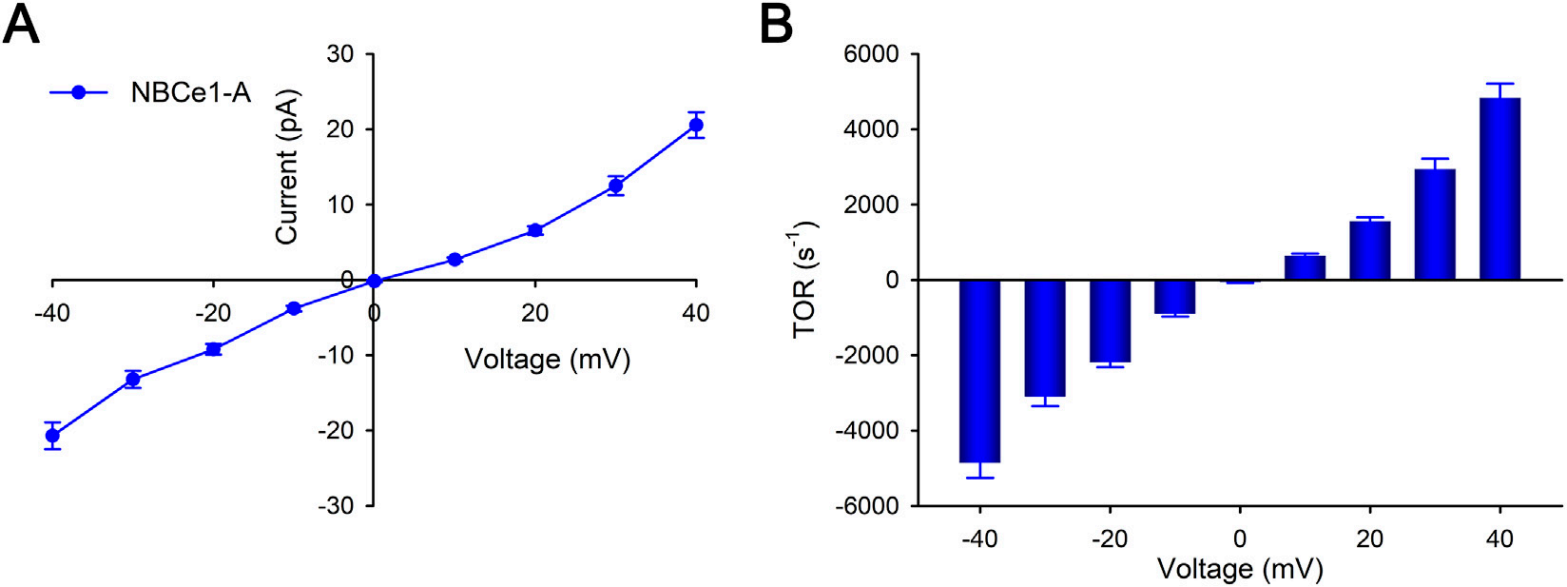
Whole cell patch clamp measurements of NBCe1-A transport currents. The effect of membrane voltage driving force *per se* in the absence of Na^+^ and CO_3_^2−^ gradients was determined. The Na^+^ concentration in both the pipet and bath was 119 mM and the estimated CO_3_^2−^ concentration was 36.7 μM. Separate cells were studied using one of the following voltage steps for each: −40 to 0 to +40 mV; −30 to 0 to +30 mV; −20 to 0 to +20 mV; −10 to 0 to +10 mV. For each voltage step protocol, a total of 6–12 separate cells were studied. **(A)** NBCe1-A transport currents were measured in the absence and presence of tenidap (paired fashion). Depicted are the tenidap-inhibitable transport currents (mean ± SEM). **(B)** NBCe1-A calculated mean TOR values at various membrane voltages are depicted.

To determine the quantitative importance of the independent effect of membrane voltage *per se* on NBCe1-A mediated currents and TOR values, the transport current was measured in the absence of Na^+^ and CO_3_^2−^ gradients. Figures 4A, B shows the dependence of the transport current and calculated TOR values on the membrane voltage respectively. The results show that at zero membrane voltage, the TOR value is 0 mV as would be expected in the absence of transported ion gradients. Moreover, TOR values are essentially independent of the absolute sign of the membrane voltage indicating that at a given absolute membrane voltage, the TOR value is independent of the direction of the NBCe1-A transport current (inward: <0 mV; outward >0 mV). In these experiments the NBCe1-A slope conductance (−40 to +40 mV) was 0.47 ± 0.02 nS with an apparent TOR-membrane voltage dependence (TOR per mV) of 109 ± 5.15 s^−1^·mV^−1^.

### 3.5 NBCe1-A OF and IF conformational states

**FIGURE 5:**
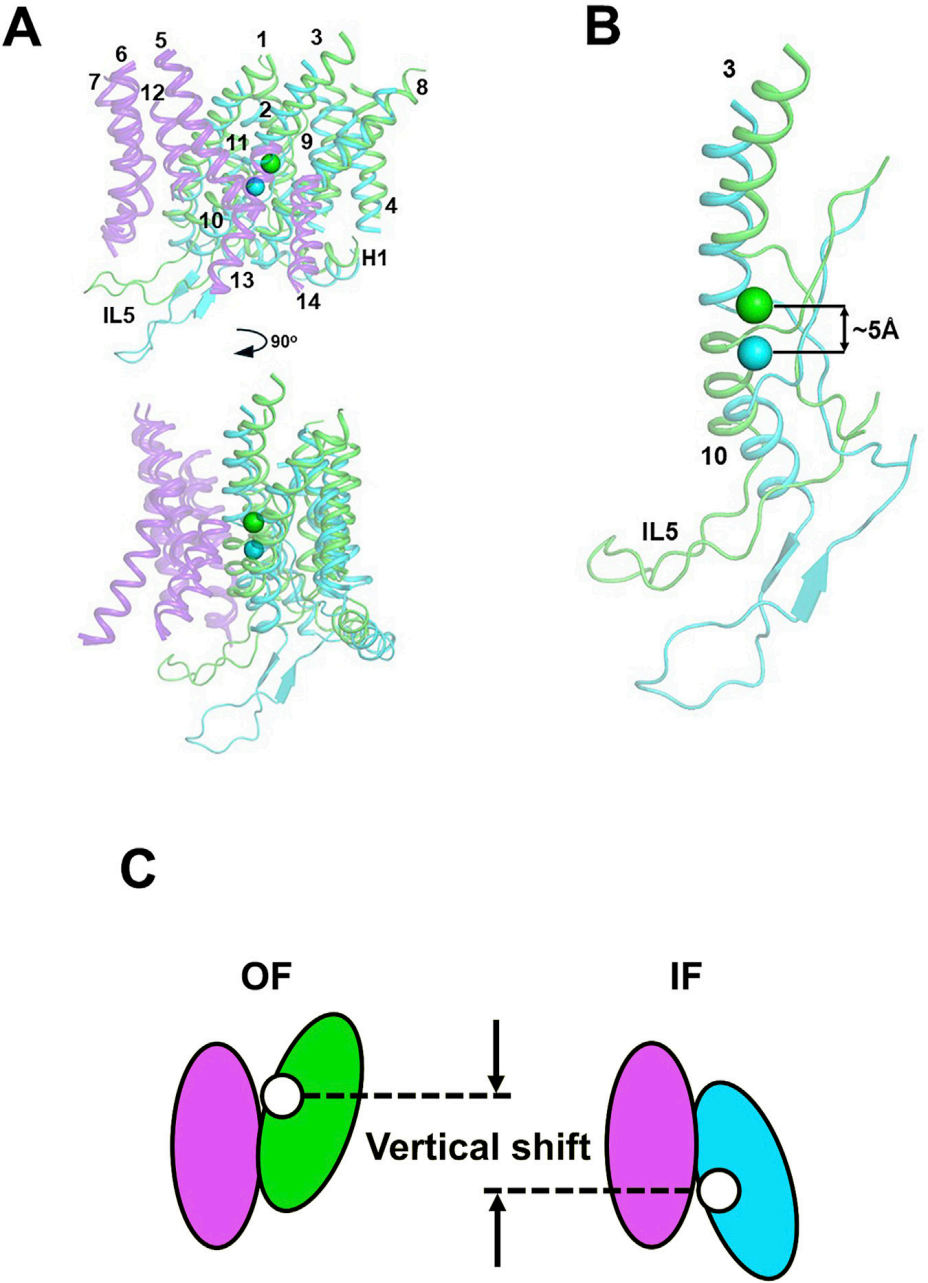
**(A)** Front and side views of the transmembrane helices of OF (green helices) and IF (cyan helices) states of NBCe1-A aligned via their gate domains (purple helices). The numbering of the TMs is also provided. All loops, except intracellular loop 5 (IL5) between TMs 10 and 11 have been removed for clarity. The center-of-mass of the backbone atoms of the residues from the ion coordination site (site S1) is shown as a green sphere (OF COM_S1_) or a cyan sphere (IF COM_S1_). **(B)** The catalytically relevant TMs 3 and 10 in the OF and IF states of NBCe1-A with their respective COM_S1_. The vertical shifts of ∼5 Å between the two COM_S1_ is indicated on the figure. **(C)** Schematic presentation of the vertical displacement. The gate domain that does not change its position during the transport cycle is shown in purple. The core domain is shown in green and blue in the OF and IF conformations respectively. The position of site S1 is indicated with a white sphere.

Figure 5 presents the overlap of the OF structure of NBCe1-A with our IF NBCe1-A conformation model. Alignment of both structures is done via their rigid gate domain, which remains relatively unchanged during the OF to IF transition, as evidenced by our recent cryoEM structures of IF-IF and IF-OF bovine AE1 dimers (Zhekova et al., 2022). Similar to the bovine AE1 structures, the NBCe1 core domain undergoes a small rotation and a vertical displacement with respect to the gate, which leads to a ∼5 Å vertical motion of the residues from site S1, identified previously in several members of the SLC4 family (Wang et al., 2021; Zhekova et al., 2021). This vertical displacement is highlighted in Figure 5 via the distance between the center-of-mass (COM_S1_) calculated in the OF (green sphere) and IF (cyan sphere) state from the backbone atoms of site S1. The 5 Å displacement is sufficient for alternate exposure of the binding site to the extra- and intracellular solution through the wide and well hydrated OF and IF cavities, formed by the residues in TMs 5, 12, 13, and 14 from the gate and TMs 1, 3, 8, and 10 from the core domain (Wang et al., 2021; Zhekova et al., 2021; Zhekova et al., 2022). The rigid motion of the core domain with respect to the gate domain and the small overall magnitude of the OF↔IF conformational change in NBCe1-A and eAE1, would require a very small reorganization of the protein structure and, as an extension, of the membrane around it. This is expected to introduce small and easily surmountable energetic barriers for the OF↔IF transition and could potentially explain the observed very high NBCe1-A and AE1 TOR numbers.

## 4 Discussion

In this study we determined the TOR values of NBCe1-A, kAE1 and eAE1. Our findings demonstrate that among SLC4 transporters, whether a given transporter is a symporter or exchanger, their TOR values are consistently high and exceed the TOR values of transporters from other families (Table 4). Using pH_i_ base flux measurements, we confirmed that eAE1 (as previously reported using Cl^−^ self-exchange (Brahm, 1977)) has a very high TOR value (present study ∼60,500 s^−1^). The N-terminal kidney-specific AE1 splice variant, kAE1, had a similar high TOR of ∼62,000 s^−1^, demonstrating that the N-terminus does not have a significant effect on the transport rate. Both anion exchangers had TOR values that were ∼2 times that of NBCe1-A (TOR value ∼30,400 s^−1^ as measured using pH_i_ base flux assays).

**TABLE 4:** Elevator-type transporters ion coordination site (ICS) vertical shift and TOR values from current study and literature.

Transporter	OF PDB	IF PDB	Vertical ICS (Å)	TOR (s^−1^)	Km/K_0.5_ (mM)	Family	References
eAE1 ghosts dimer	4YZF	ND	ND	50,000	HCO_3_^‾^: 10 Cl^‾^: 30	SLC4	Brahm (1977), Macara and Canttley (1981b), Wieth (1979), Gasbjerg et al. (1996), Lepke (1976)
eAE1 HEK293 dimer				60,491			Current study
kAE1 HEK293 dimer				62,241			Current study
Bovine AE1 dimer	8E34	8D9N	5.0	ND	ND	SLC4	Zhekova et al. (2022)
Human NBCe1 dimer	6CAA	IF model current study	5.0	30,422	HCO_3_^‾^: 10 Na^+^: 30	SLC4	Huynh et al. (2018) Current study
Human AE2 dimer	8GVF	8GVF	5.0*	ND	Cl^‾^ external 5.6	SLC4	Humphreys et al. (1994), Zhang et al. (2023)
SeCitS dimer	5A1S	5A1S	15.2	0.02	Citrate: 0.0041 Na^+^(2): 3.3	2HCT	Wöhlert et al. (2015)
KpCitS dimer	5X9R 5XAS	5XAT 5XAS	13.9	137	Citrate: internal: 13.5 external: 0.14	2HCT	Pos and Dimroth (1996), Kebbel et al. (2013), Wöhlert et al. (2015), Kim et al. (2017)
TtNapA dimer	5BZ3	5BZ2	8.6	400–435, 1,433	ND	Na^+^/H^+^-antiporters	Taglicht et al. (1991), Appel et al. (2009), Cha et al. (2009), Lee et al. (2013), Lee et al. (2014), Coincon et al. (2016)
EcNhaA dimer	ND	4ATV	ND	1,500	Na^+^: 0.5	Na^+^/H^+^-antiporters	Appel et al. (2009), Lee et al. (2013), Lee et al. (2014)
BicA, dimer	ND	6KI1 6KI2	6.0	ND	Na^+^: 1.7 HCO_3_^‾^: 0.074–0.353	SLC26	Price et al. (2008), Wang et al. (2019)
Mouse Slc26a9 dimer	ND	6RTC 6RTF	ND	ND	Cl^‾^: 30 SCN^‾^: 0.5	SLC26	Walter et al. (2019)
CNT_NW_ trimer	512A 512B	5,126	7.8	9.6, 8.3–181	Uridine: 0.022 0.130	SLC28	Smith et al. (2004), Hirschi et al. (2017)
ASCT2 trimer	6MP6	6GCT	18.7	65	Na^+^(1): 0.3; Na^+^(2): 14–27	SLC1	Garaeva et al. (2018), Yu et al. (2019), Wang et al. (2022)

**FIGURE 6:**
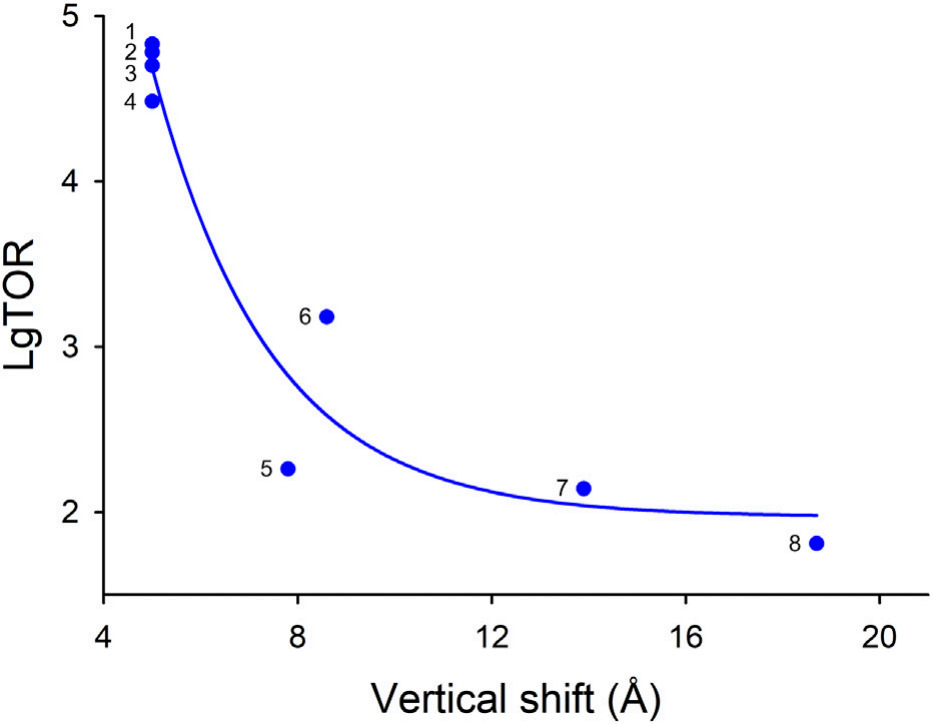
Apparent dependence of various elevator-type transporter TOR values on their substrate coordination site vertical shift that occurs during the OF to IF transport cycle conformation changes. If several TOR numbers were available in the literature for a given transporter, the largest value was used in this analysis. The TOR numbers and vertical shift data are provided in Table 4. Transporters: (1) kAE1; (2) eAE1 (human); (3) eAE1 (bovine); (4) NBCe1-A; (5) CNTnw; (6) TtNapA; (7) KpCitS; (8) ASCT2.

AE2 vertical ion coordination site (ICS) shift was calculated in the current study based on AE2 OF, and IF, conformational structures (Zhang et al., 2023); ND: no data. We did not use SeCitS data in Figure 6 because of the extremely low TOR, number that distinguishes it from other transporters whose TOR, have been measured including the functionally and structurally similar KpCitS transporter.

In any alternating access model, a transport event requires initially permeation of substrate(s) from an aqueous phase into the interior of the transporter, subsequent binding to the substrate coordination site, a protein conformational change exposing the substrate(s) to an aqueous phase on the opposite side of the membrane, and dissociation of the substrate(s) from the transporter. In the case of uniporters or symporters, the reverse process and associated conformational changes occur in the absence of substrate where in the context of exchangers, a different substrate is transported in the reverse direction. These events can be described in a simple Michaelis-Menten kinetic model (Vivian and Polli, 2014). Our data suggests that fast and unimpeded substrate permeation to/from the binding site and the consecutive rapid conformational structural changes are a potentially important factor when considering the high NBCe1-A and AE1 TOR values, and likely other SLC4 transporters given their shared structural fold. All known SLC4 proteins structures feature a wide, well hydrated cavity which allows swift and unobstructed permeation of the substrate ions to and from site S1 in both OF and IF states as seen in previous computation modeling studies (Zhekova et al., 2021; Zhekova et al., 2022; Wang et al., 2021). Previous OF structural data (Huynh et al., 2018) coupled with our current IF computational modeling indicates that among SLC4 transporters, NBCe1-A, eAE1, AE2 (Zhang et al., 2023) and SLC4A11 (Lu et al., 2023) utilize an elevator-type transport mechanism (Garaeva and Slotboom, 2020). In the present study, based on our previous NBCe1-A OF structural data (Huynh et al., 2018) and our current NBCe1-A IF modeling, the vertical shift of the NBCe1 ion coordination site is ∼5 Å (Figure 5) in line with the displacement determined previously in bovine AE1 (Zhekova et al., 2022). We hypothesize that the short vertical shift coupled with the small protein and membrane reorganization leads to low energetic barriers of the conformational transitions and short time involved in ion transfer between the two aqueous phases. The vertical shift of the ion coordination site of the aforementioned SLC4 proteins is ∼5 Å (Zhekova et al., 2022), which is significantly smaller than the vertical shift of most other reported elevator-type transporters, which vary from 6.0 to 18.7 Å (Table 4).

Comparison of the known elevator-type transporters, for which TOR numbers are available, suggests that there is inverse relationship between TOR number and the vertical shift of the ion coordination site between the OF and IF states during the transport cycle (Figure 6). If this relationship is generalizable to transporters such as AE2 (SLC4A2), which has a similar small ∼5 Å vertical ion coordination site shift (Zhang et al., 2023), but for which the TOR value is yet to be determined, it can be predicted that AE2 would also have an unusually high TOR value.

In the current study, the ability to measure NBCe1-A transport electrophysiologically allowed its TOR values to be determined by assessing both its transport current and base flux (Figures 1, 3; Table 3). These separate methods demonstrated the high TOR value of NBCe1-A. In addition, the TOR-dependence on the membrane voltage *per se* was determined (Figure 4). In these experiments, the transcellular Na^+^ and CO_3_^2−^ gradients were zero such that a change in the membrane potential was the only thermodynamic factor determining the direction of transport (Beckstein and Naughton, 2022). As shown in Figure 4, the NBCe1-A TOR values measured at the same absolute membrane voltage (positive versus negative sign) did not differ significantly. These findings demonstrated that over the range of membrane voltages studied, the TOR numbers were independent of the direction of the transport current (i.e., pipet to bath versus bath to pipet). Whether this property of NBCe1-A is generalizable to other SLC4 and non-SLC4 transporters requires further study. Interestingly, the TOR values of the EAAC1 glutamate transporter differ from NBCe1-A in being strongly transport direction dependent (Zhang et al., 2007).

Mutations in NBCe1 and kAE1 cause proximal and distal renal tubular acidosis, respectively (Kurtz, 2018; Wagner et al., 2023). In distal renal tubular acidosis, the transport of HCO_3_‾ in the type A intercalated cells in the collecting duct is impaired. Our findings indicate that under similar experimental conditions when expressed in HEK293 cells, kAE1 and eAE1 have similarly high TOR values. Under the conditions of this study the intracellular milieu was essentially identical. However, this is the not the case *in vivo* where the N-terminal binding proteins differ between red cells expressing eAE1 and type A intercalated cells expressing kAE1. In this regard, it is unknown whether these additional factors can alter their respective *in vivo* TOR values.

In a simple transport kinetic scheme (Vivian and Polli, 2014), the K_m_ depends on each of the rate constants for the following transport steps that occur bidirectionally: 1) substrate binding to the transporter, 2) conformational change of transporter with the bound substrate, 3) release of the substrate, and finally 4) the conformational change in the reverse direction to complete the cycle. Vivian and Polli (Vivian and Polli, 2014) demonstrated that high transport rates were associated with high K_m_ values and mostly dependent on the rate constant of the protein conformational changes. Accordingly, mutations in the human copper transporter 1 (hCTR1) that increased the K_m_, were accompanied by increases in TOR numbers (Maryon et al., 2013). In keeping with this finding, OCT2, which has a higher half-saturation constant (K_t_) for metformin (∼300 µM) than for 1-methyl-4-phenylpyridinium (∼5 µM), had a higher maximal transport rate for metformin (300 pmol·min^−1^·cm^−2^) than for 1-methyl-4-phenylpyridinium (30 pmol·min^−1^·cm^−2^) (Severance et al., 2017). Accordingly, it would be of interest to determine to what extent the substrate K_m_ values for eAE1 (Brahm, 1977; Wieth, 1979) and NBCe1-A (Sciortino and Romero, 1999; Grichtchenko et al., 2000) affect the rate constants of their proposed rapid elevator-like conformational changes during the transport cycle.

Structurally, SLC4 transporters are arranged in a 7+7 TM inverted repeat fold, in which TMs 8–14 sequences resemble the inverted sequences of TMs 1–7. The other known transporters whose TOR value has been measured and share an elevator-like transport mechanism do not have a 7+7 TM inverted repeat fold. A 7+7 TM inverted repeat fold is also utilized by the anion transporting SLC26 transporters including bacterial SLC26Dg (Geertsma et al., 2015), mouse SLC26A9 (Walter et al., 2019) and gerbil SLC26A5 (Butan et al., 2022) and the SLC23 transporters including the fungus purine symporter UapA (Alguel et al., 2016) and the bacterial uracil transporter UraA (Lu et al., 2011). Given that no TOR values for these transporters are available, it is not yet possible to determine whether all 7+7 inverted repeat transporters possess high TOR numbers, or whether this is a unique attribute of the SLC4 transporters. Future studies will address this important question.

## Data Availability

The raw data supporting the conclusions of this article will be made available by the authors, without undue reservation.
